# Endometrial Polyps in Women Affected by Levothyroxine-Treated Hypothyroidism—Histological Features, Immunohistochemical Findings, and Possible Explanation of Etiopathogenic Mechanism: A Pilot Study

**DOI:** 10.1155/2013/503419

**Published:** 2013-08-22

**Authors:** Carlo Saccardi, Salvatore Gizzo, Kathrin Ludwig, Maria Guido, Mara Scarton, Michele Gangemi, Raffaele Tinelli, Pietro Salvatore Litta

**Affiliations:** ^1^Department of Women's and Children's Health, University of Padua, 35128 Padua, Italy; ^2^Department of Medical Diagnostic and Special Therapy, University of Padua, 35128 Padua, Italy; ^3^Ospedale San Bassiano, OB/GYN Unit, 36061 Bassano del Grappa, Vicenza, Italy

## Abstract

The aim of the study was to investigate the possible overexpression of estrogen (ERs) and progesterone (PRs) receptors both in EPs glandular and stromal cells in postmenopausal women with levothyroxine-treated hypothyroidism in comparison to EPs detected in women with physiological thyroid hormone levels. During the study period (January-February 2013) 22 patients were eligible (12 treated, 10 controls). The two groups were homogenous for general, EPs sonographic and hysteroscopic features. None of the cases of atypia was found. Immunohistochemistry showed that the two groups were similar for ERs and PRs intensity rates in EPs glandular cells despite a trend of ERs percentage expression more than 60% in 2/3 of treated patients versus 1/3 of controls. In stromal EPs components, ERs intensity was high positive in 10 (83,3%) treated cases while it was high positive in 1 control (10%). Percentage of ERs stromal expression showed a different trend between the two groups despite a borderline statistical significance. Our hypothesis is based on a possible double action of hypothyroidism and thyroxine intake: the subclinical TSH increased levels and its possible circadian oscillation could stimulate the endometrial TSHRs (increasing type 2 DIO activity); the circulating levels of exogenous thyroxine could be locally metabolized in active form by type 2 DIO stimulating ERs.

## 1. Introduction

Endometrial polyps (EPs) are one of the most common gynecological conditions often diagnosed incidentally or associated with clinical symptoms such as infertility in reproductive age and abnormal vaginal bleeding both in premenopausal and postmenopausal women  [1]. 

The real incidence of EPs is unknown since endometrial polyps can occur in symptom-free women; therefore the reported prevalence varies widely and ranges from 7.8% to 34.9%, depending on the definition of a polyp, diagnostic method used, and the population studied  [2, 3].

The prevalence of EPs appears to increase with age, and it is reported that more postmenopausal (11.8%) than premenopausal women (5.8%) are affected  [1].

The most frequent treatment of EPs is hysteroscopic removal of the lesions that could be performed both with anaesthesia in day-surgical setting and with office approach without anaesthesia  [4].

Many authors have conducted studies on risk factors for EPs onset both, in pre- and postmenopause. In most of these studies age and unopposed estrogen stimulation resulted in independent risk factors for EPs development  [5–7]. 

A relationship between estrogens, increased expression of endometrial hormonal receptors, (HRs) and endometrial stimulation is already accepted [8, 9]. In the last years it has been suggested that estrogens can cause the EPs growth by failure of proapoptotic mechanisms (an overexpression of Bcl-2 was reported in premenopausal and postmenopausal endometria) as well as by an increase in local levels of growth factors (GFs) such as fibroblastic GF, transforming GF-a, epithelial GF, IGF-1, and GF receptors within the endometrium which may promote polyp growth  [5, 8]. 

Even if many studies have been conducted on the relation between thyroid hormones (THs), thyroid hormone receptors (THRs), and implications on endometrial function such as fertility and implantation outcomes  [10, 11], no studies have been conducted on the influence of THs dysregulation in EPs onset.

In our clinical practice we observed that levothyroxine-treated postmenopausal women with hypothyroidism, often referred to hysteroscopy investigation after ultrasound suspicious of large EPs.

The aim of the study was to investigate the possible overexpression of estrogen (ERs) and progesterone (PRs) receptors both in glandular and stromal cells of EPs in postmenopausal women with levothyroxine-treated hypothyroidism in comparison to EPs detected in women with normal physiological thyroid hormone levels.

## 2. Materials and Methods 

In interval time between January and February 2013 in Obstetrics and Gynecological Clinic of Padua we conducted a pilot case-control study on cohort of asymptomatic postmenopausal women referred to hysteroscopy for suspicious of large endometrial polyps with ultrasound estimated size more than 2 cm.

All enrolled patients were properly informed about the aim of the study, and they consented in a written consent form describing the use of their privacy data (Italian law 675/96), respecting also the regulations about the studies on human tissue of the Institute of Pathology of the University of Padua.

The inclusion criteria were personal and gynecological history negative for major medical conditions (diabetes, hypertension, obesity, and cancer) except for hypothyroidism treated by levothyroxine, diagnosis of physiological menopause since at least two years, no history of previous or concomitant use of hormone replacement therapy and hormonal adjuvant therapy, no history of abnormal uterine bleeding, and transvaginal ultrasound investigation performed within no more than 30 days prior to hysteroscopy.

For all patients the following laboratory and clinical parameters were evaluated: age, weight, BMI, parity, sonographical and hysteroscopic size of the EPs, and TSH serum levels before polypectomy. For the patients with levothyroxine-treated hypothyroidism we furthermore considered dosage and duration of treatment. 

All patients received an office hysteroscopy with a continuous-flow hysteroscope (Karl Storz—Karl Storz GmbH & Co., Tuttlingen, Germany) using saline solution (0.9% sodium chloride, pH 5.5) as a distension medium and 30° angle view optics (2.9 mm diameter) in order to confirm the ultrasound suspect of EPs and evaluate the cervical and uterine cavity.

All patients had a subsequent operative hysteroscopy under anesthesia in operating room through operative resectoscope of 10 mm diameter, (Karl Storz—Karl Storz GmbH & Co., Tuttlingen, Germany) using monopolar loop and hypotonic distension medium (1% glycine, 1% mannitol in 1000 mL of water).

All removed EPs were sent to histological examination.

### 2.1. Histology and Immunohistochemistry

All samples were immediately fixed in 10% buffered formalin and embedded in paraffin. Serial histological sections 4–6 *μ*m thick were obtained from each paraffin block selected. The histological sections were stained with hematoxylin and eosin. The diagnosis was confirmed by two pathologists in all cases (M.G.; Kathrin Ludwig). Immunohistochemical staining was done automatically (Bond-Max Immunostainer, Menarini Diagnostics, Firenze, Italy) for PR (clone 16, Novocastra Laboratories Ltd., Newcastle Upon Tyne, UK; 1 : 100) and ER (clone 6F11, Novocastra Laboratories Ltd., Newcastle Upon Tyne, UK; 1 : 50) according to the manufacturers' instructions. 

Appropriate positive and negative controls were run concurrently.

PR and ER nuclear and cytoplasmic expression was jointly scored by two pathologists (Maria Guido and Kathrin Ludwig) unaware of the patients' clinical history. Nuclear and cytoplasmic ER and PR staining was scored on a four-tiered scale (score 0, ≤5% positive nuclear/cytoplasmic staining; score 1, ≥5 ≤30% positive nuclear/cytoplasmic staining; score 2, >30% ≤60% positive nuclear/cytoplasmic staining; and score 3, ≥71% positive nuclear/cytoplasmic staining; all evaluated on 10 consecutive “*high power fields*”) and subsequently classified as *low *or* high*.

### 2.2. Statistical Analysis

Statistical analysis was performed using SPSS statistical software (Chicago, IL) version 19 for Windows. The results were expressed in absolute numbers and percentages for discrete variables and in average ± standard deviation for continuous variables.

We performed appropriate parametric and nonparametric statistical tests when possible using the Kolmogorov-Smirnov test as a normal distribution of the sample. Continuous variables were analysed by *t*-test, and categorical variables were analysed by the *χ* ^2^ test or Fisher's exact test. *P* values of *P* < 0.05 were considered statistically significant.

## 3. Results

In the interval time considered 52 patients agreed to participate in the study but only 22 were eligible.

Of these, 12 women had a diagnosis of hypothyroidism and had been treated with levothyroxine for at least one year (Group A); the remaining 10 women had a normal thyroid function (Group B).

The results of two groups were homogeneous for general features (Table 1).

A comparison of Group A versus Group B in terms of EPs sonographic size showed no difference. Similarly, data about EPs hysteroscopic size showed no difference between the groups despite the fact that we found a mean hysteroscopic size value bigger than the mean sonographic size (Table 1).

Group A patients resulted levothyroxine users from an average time of 21.4 ± 11.7 years (range 4–37 years). Concerning levothyroxine dosage, we found that 5 patients took a mean dose of 50 ugr/die (41.7%), 5 patients took a mean dose of 75 ugr/die (41.7%), and finally only 2 patients took a mean dose of 100 ugr/die (16.6%).

**Figure 1:**
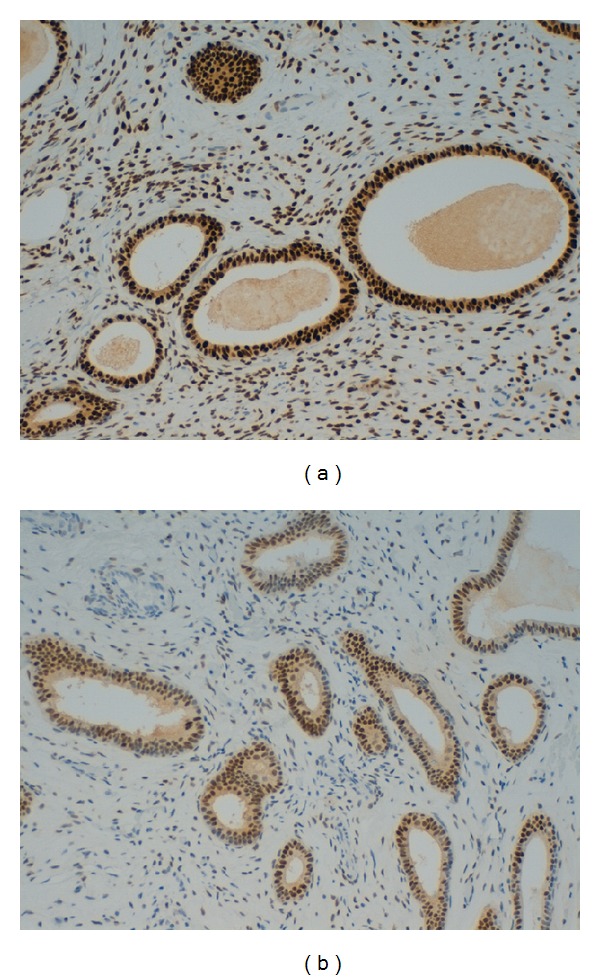
Immunohistochemical findings of estrogen receptors expression in endometrial polyps: (a) polyps from levothyroxine-treated patients and (b) polyps from control group (original magnification 20x).

Data about immunohistochemical findings showed that the two groups were similar for ERs and PRs intensity rates in EPs glandular cells *(P: n.s.)* despite the fact that we found a trend of ERs percentage expression more than 60% in 2/3 of Group A EPs versus 1/3 of Group B EPs (Table 2; Figure 1).

Concerning stromal EPs components, ERs intensity was low positive in 2 cases (16.7%) and high positive in 10 (83.3%) cases of Group A while in Group B it was low positive in 9 cases (90%) and high positive in 1 case (10%) *(P: 0.02) *(Table 2).

Data about percentage of ERs stromal expression showed a different trend between the two groups despite a borderline statistical significance: 1 case (8.3%) with 5–30% positivity, 9 cases (75%) with 30–60% positivity, and 2 cases (16.7%) with more than 60% positivity were detected in Group A while in Group B there were 5 cases (50%) with 5–30% positivity, 5 cases (50%) with 30–60% positivity, and none with more than 60% positivity *(P: 0.05)* (Table 2).

Immunohistochemical findings are shown in Figure 1.

Concerning histological features of EPs, in Group A, 4 were functional and 8 atrophic, while in Group B, 3 were functional and the remaining 7 were atrophic. None of the EPs analyzed were positive for atypia at microscopic histological examination.

## 4. Discussion

Nowadays many pieces evidence reported that thyroid hormones play a role in various aspects of human reproduction since both hypothyroidism and hyperthyroidism seem to have implications on sex steroids metabolism and ovarian and endometrial functions in women leading to reproductive disorders such as menstrual irregularities and infertility  [12].

Even if the suspects about THs effects on endometrium are dated, the confirmation of the expression and distribution of TRs, TSHRs, and iodothyronine deiodinase (DIO) in human endometrium were obtained firstly only in 2011  [12].

Aghajanova et al. demonstrated also that THs could be synthesized by both endometrial stromal cell and Ishikawa cell cultures after TSH stimulation  [12]. This evidence suggested the hypothesis that endometrial stromal cells have TSHRs capable of binding thyrotropin, thus initiating secretion of THs independently from the hypothalamo-pituitary-thyroid system.

This concept was hypothesized by Catalano et al. who demonstrated that progesterone molecular pathways are implicated in endometrial THs metabolism and signalling such as type II DIO, thyroid peroxidase (TPO), and thyroglobulin (TG). It is interesting that both cyclic changes of type II DIO and TRs expression in human endometrium showed an inverse relationship with progesterone levels confirming the hypothesis of progesterone influence  [13].

Even if the debate about the role of estrogen and progesterone in the pathophysiology of EPs is still unresolved, the elongation of endometrial glands, stromal tissues, and spiral arteries that give the characteristic polypoid appearance of endometrium could be considered the most frequent features detected in case of local hormonal disorders.

In postmenopausal women, estrogen receptors are present in higher number of polyps than in normal endometrium  [14], while only a limited evidence shows that progesterone receptors may contribute, only by themselves, to polyp development in some women  [15].

A previous immunohistochemical study, according to our evidence, reported a stronger ERs and PRs expressions in glandular epithelium of polyps than in glandular epithelium of adjacent endometrium. A stronger ERs expression in the stroma of polyps than in stroma of adjacent endometrium was also reported. Authors concluded that in the pathogenesis of EPs a trophic paracrine interaction between stromal and epithelial cells, through the induction of epithelial estrogen proliferation via stromal estrogen receptors, represents the more logical mechanism  [14].

Although no evidence is demonstrated directly in human endometrium, the relationship between THs and estrogen actions has been documented in many physiological functions  [16].

Both hormones stimulate transcription of target genes binding to their nuclear receptors that interact with specific responsive elements (estrogen and thyroid hormone response elements, i.e., ERE and TRE, resp.) in the regulatory regions of the gene. 

In vitro studies have suggested that an interplay between the two hormones might be due to cross-talk at hormone responsive elements, with the respective hormone receptors and ligands being able to interact and induce similar cell cycle progression and proliferation of both p53 and pRb mediated. Thyroid hormone has been demonstrated both to potentiate and to inhibit estrogen-induced gene expression. A possible hypothesis is that TR and ER bind two parts of the same hormone response elements of a target gene and can compete with each other. In this way, ER and TR interactions would be either potentiating or mutually inhibitory depending on the absence or presence of ligands, on the affinity for the receptor elements, and on the consecutive protein-DNA interactions  [17].

The most accredited theory proposed a simpler mechanism for THs effects on estrogen responses through the increase in *α*ERs with resultant increase in PRs, prolactin production and tumor growth  [16].

The results of our pilot study, even if obtained in a small number of cases, confirm the expected different ERs pathway in postmenopausal women with large benign EPs linked to hypothyroidism and THs replacement therapy.

A limitation of our study could be that we considered only women affected by EPs; we did not perform a peripheral endometrial biopsy; we considered only menopausal women; and, finally, the small size of the sample did not allow to relate dosage and length of levothyroxine treatment to cells findings.

## 5. Conclusion

Since our initial hypothesis was confirmed, we will start a perspective large cohort study in order to detect detailed different hormonal pathways in EPs pathogenesis between postmenopausal women levothyroxine-treated affected by hypothyroidism and controls.

We will investigate stromal and glandular ERs and PRs expressions in EPs, peripheral and distant endometria in respect to polypoid tissue.

We will also investigate, by immunohistochemical analysis, the expression of TSHRs, type II DIO activity, TR*α*1, TR*α*2, TR1*β*, VEGF, TGF *β*, and p53 in EPs, and adjacent endometrium in order to elucidate the molecular mechanism through hypothyroidism could induce benign EPs.

This hypothesis is based on a possible double action of hypothyroidism and thyroxine intake: on one hand the subclinical TSH increased levels and its possible circadian oscillation linked to levothyroxine pharmacokinetic could stimulate the endometrial TSHRs (increasing type 2 DIO activity); on the other hand the circulating levels of exogenous thyroxine could be locally metabolized in active form by type 2 DIO stimulating ERs (especially stromal ones) simulating the action of estrogens. 

The absence of glandular and stromal atypia detection during histological investigation of EPs proliferating by this mechanism could be related to high levels of PRs expression induced by ERs activity similar to the mechanism of onset of EPs detected in premenopausal women.

## Figures and Tables

**Table 1:** General patients features: Group A (treatment) versus Group B (controls).

Variables	Groups (number)	Mean (± standard deviation)	Range
Age (years)	Group A (12)	60.6 ± 3.39	56–65
	Group B (10)	63.2 ± 3.39	57–68

Weight (kg)	Group A (12)	58.7 ± 3.59	55–64
	Group B (10)	61.0 ± 3.33	55–65

BMI	Group A (12)	28.0 ± 1.62	26–30
	Group B (10)	27.0 ± 1.49	25–30

Parity	Group A (12)	2.1 ± 1.8	0–4
	Group B (10)	2.4 ± 1.3	0–5

Polyps sonographic size (cm)	Group A (12)	2.3 ± 0.19	2.1–2.7
	Group B (10)	2.3 ± 0.21	2.1–2.6

Polyps hysteroscopic size (cm)	Group A (12)	2.5 ± 0.19	2.5–3.0
	Group B (10)	2.6 ± 0.21	2.5–3.0

Preoperative TSH serum level (UI/L)	Group A (12)	2.2 ± 0.6	1.1–3.2
	Group B (10)	2.5 ± 0.5	1.5–3.2

**Table 2:** Immunohistochemical endometrial polyps features: Group A (treatment) versus Group B (controls).

Group	Total number	Stromal ERs intensity	Number (%)	*P*	Glandular ERs intensity	Number (%)	*P*
A	12	Low	2 (16.7)		Low	2 (16.7)	0.05
		High	10 (83.3)	0.02	High	10 (83.3)	
B	10	Low	9 (90)		Low	4 (40)	
		High	1 (10)		High	6 (60)	

Group	Total number	Percentage of stromal ERs	Number (%)	*P*	Percentage of glandular ERs	Number (%)	*P*

A	12	5–30%	1 (8.3)		5–30%	0	n.s.
		30–60%	9 (75)		30–60%	4 (33.3)	
		>60%	2 (16.7)	n.s.	>60%	8 (66.7)	
B	10	5–30%	5 (50)		5–30%	0	
		30–60%	5 (50)		30–60%	7 (70)	
		>60%	0 (0)		>60%	3 (30)	

Group	Total number	Stromal PRs intensity	Number (%)	*P*	Glandular PRs intensity	Number (%)	*P*

A	12	Low	1 (8.3)		Low	0 (0)	n.s.
		High	11 (91.7)	n.s.	High	12 (100)	
B	10	Low	1 (10)		Low	(0)	
		High	9 (90)		High	10 (100)	

Group	Total number	Percentage of stromal PRs	Number (%)	*P*	Percentage of glandular PRs	Number (%)	*P*

A	12	5–30%	1 (8.3)		5–30%	0 (0)	n.s.
		30–60%	4 (33.3)		30–60%	1 (8.3)	
		>60%	7 (58.3)	n.s.	>60%	11 (91.7)	
B	10	5–30%	0 (0)		5–30%	0 (0)	
		30–60%	7 (70)		30–60%	2 (20)	
		>60%	3 (30)		>60%	8 (80)	

ERs: estrogen receptors; PRs: progesterone receptors.
